# Different Neural Correlates of Automatic Emotion Regulation at Implicit and Explicit Perceptual Level: A Functional Magnetic Resonance Imaging Study

**DOI:** 10.1177/2041669519831028

**Published:** 2019-02-27

**Authors:** Yu Xie, Zhiguo Hu, Weina Ma, Biao Sang, Mian Wang

**Affiliations:** Department of Psychology, Faculty of Education, Hangzhou Normal University, China; Department of Special Education, Faculty of Education, East China Normal University, Shanghai, China; Department of Psychology, Faculty of Education, Hangzhou Normal University, China; Center for Cognition and Brain Disorders, Hangzhou Normal University, China; Institutes of Psychological Sciences, Hangzhou Normal University, China; Department of Psychology, Faculty of Education, Hangzhou Normal University, China; Institutes of Psychological Sciences, Hangzhou Normal University, China; The School of Psychology and Cognitive Science, East China Normal University, Shanghai, China; Gevirtz Graduate School of Education, University of California, Santa Barbara, CA, USA

**Keywords:** automatic emotion regulation, implicit, explicit, perceptual level, functional magnetic resonance imaging

## Abstract

Automatic emotion regulation (AER) is an important type of emotion regulation in our daily life. Most of the previous studies concerning AER are done in the conscious level. Little is known about the AER under the subliminal level. The present study was to investigate the AER at the different perceptual levels (i.e., explicitly and implicitly) simultaneously, and the associated neural differences using functional magnetic resonance imaging. Priming paradigm was adopted in which the inhibition or neutral words were used as primes and the negative picutres were used as targets. In the experiment, the duration time of priming words was manipulated at 33 or 50 ms in the implicit level and 3000 ms in the explicit level. The participants were required to make emotional valence rating of the negative pictures while undergoing functional magnetic resonance imaging scanning. The results showed that the participants experienced less negative emotion in inhibition words priming condition contrary to neutral words priming condition. Significant differences were also found in the left ventrolateral prefrontal cortex and left dorsolateral prefrontal cortex at the implicit and explicit AER. The findings of this study demonstrate that inhibition words can automatically and effectively reduce an individual’s negative emotion experience, and left ventrolateral prefrontal cortex and left dorsolateral prefrontal cortex have been both implicated in self-control during AER.

## Introduction

Automatic emotion regulation (AER) is one of the most common types of emotion regulation in our daily life. Overlearned habits (Aarts & Dijksterhuis, 2000), regulatory strategies learned early in childhood (Mauss, Bunge, & Gross, 2007), sociocultural norms (Adams & Markus, 2004), and implicit hedonic goals (Koole & Jostmann, 2004; Rudman, 2004) can all develop into automatic regulatory processes. AER changes the emotional trajectory with automatic pursuit of goals and can deal with some life problems that resist controlled regulation. According to Mauss et al. (2007), AER is a goal-driven change to any aspect of one’s emotions without making a conscious decision to do so, without paying attention to the process of regulating one’s emotions, and without engaging in deliberate control. Thus, it can be independent of cognitive resource constraints (Carstensen & Mikels, 2005), flexibly adjust the mood (Koole & Coenen, 2007; Koole & Jostmann, 2004), improve the adaptive ability, and reduce the risk of emotional disorders.

AER recruits a sophisticated neural network. Phillips, Ladouceur, and Drevets (2008) proposed a neural model of AER, including bilateral anterior cingulated cortex (ACC), bilateral orbitofrontal cortex (OFC), dorsomedial prefrontal cortex, hippocampus, and parahippocampus. It is noted that in the current neuroscientific research literature, there are different experimental paradigms employed to provide empirical evidence for the earlier model. These experimental paradigms consist of the emotional conflict paradigm (Etkin, Egner, Peraza, Kandel, & Hirsch, 2006; Haas, Omura, Constable, & Canli, 2006), the emotion regulation by implementation intentions (Hallam et al., 2015), and the affect labeling paradigm (Lieberman et al., 2007). The emotional conflict paradigm refers to experimental situations where a cognitive conflict is set up for the subject by presenting emotional words (e.g., *happy*) overlaid on emotional faces (e.g., fearful face), and then the subject is asked to name the emotional facial expression (e.g., happy or fear; Etkin et al., 2006) or judge the emotional valence of the words (Haas et al., 2006). Numerous studies have adopted the emotional conflict paradigm to investigate the neural mechanisms of emotional regulation which led to a set of important findings: a regulatory interaction between the ACC and the medial prefrontal cortex (mPFC; Etkin et al., 2006; Etkin, Prater, Hoeft, Menon, & Schatzberg, 2010), the involvement of dorsal ACC and mPFC in appraisal and expression of negative emotion, as well as the ventral–rostral portions of the ACC and mPFC in generating emotional responses (Etkin, Egner, & Kalisch, 2011). As for the emotion regulatory intention paradigm, Hallam et al. (2015) conducted an experimental study to investigate the neural basis of AER involved in implementation intention. It is found that while the participants engaged in the pictures rating task by utilizing previously taught implementation intention strategies (e.g., “if I see a snake, then I will think there are just pixels on the screen!”), the functional magnetic resonance imaging (fMRI) results revealed the activation of right inferior frontal gyrus and ventro-parietal cortex during AER. Other studies also pointed to the role of medial OFC in AER with respect to implementation intention tasks (Etkin et al., 2011). While Lieberman et al. (2007) suggested that emotion regulation in affect labeling paradigm (e.g., participants were instructed to decide whether the facial expression was angry or happy) is implicit, it might be the mPFC-amygdala pathway that plays a critical role in emotion regulation process.

Recent research has paid more attention to the role of ventrolateral prefrontal cortex (vlPFC) in AER. It has been suggested that the right vlPFC is implicated in inhibitory control (Aron, Robbins, & Poldrack, 2014) and is the implicit form of emotion control (Cohen, Berkman, & Lieberman, 2013). Yoon, Kim, and Kim (2015) speculated that nonconscious emotion regulation was mediated by the right vlPFC based on the findings of their study where the facial expression was used to prime the implicit regulation of the negative picture. However, Tupak et al. (2014) indicated that the specificity of vlPFC is to activate with respect to stimulus valence during labeling of picture task.

It appears that the previous studies concerning AER are almost all done in the conscious level. Yet little is known about the AER under the subliminal level. According to Gross and Thompson (2007), emotional regulation refers to the process of individual’s influence on the occurrence, experience, and expression of emotion, and it is a continuum from AER to voluntary emotion regulation. The purpose of this study was to investigate the AER of the negative pictures at the different perceptual levels (i.e., explicit and implicit) simultaneously, and the associated differences in the brain regions being activated.

As for the process of AER, priming technique was one of the most commonly used strategies to effectively control the implicit regulatory processes (Yang, Tang, Gu, Luo, & Luo, 2015). In a study where the priming techniques were used (Mauss, Cook, & Gross, 2007), the participants were asked to construct four-word sentences that contain emotional control terms (e.g., *restrains*). In this process, AER was successfully induced when participants were manipulating the priming emotional control words implicitly. There were two distinctive neural systems underlying the emotional perception, one operating at the explicit conscious level, and the other operating implicitly, below the consciousness level (de Gelder, Vroomen, Pourtois, & Weiskrantz, 1999; Driver, Vuilleumier, Eimer, & Rees, 2001; Whalen et al., 1998). Undoubtedly, priming materials at the different perceptual levels (either implicit or explicit) would influence AER. Moreover, “putting feelings into words” is one of the best ways to manage negative emotional experiences (Lieberman et al., 2007). We intended to adopt the priming words to arouse the participants’ implicit perception level of AER in the present study. The priming words used in this study consist of inhibition words and neutral words, which are not significantly different in the emotional valence but in the sense of control.

This study aimed to examine whether inhibition words can effectively prime AER, and how activation of brain regions associated with the tasks at implicit and explicit perceptual levels differ during the AER process by using fMRI. It was expected that inhibition word priming condition (as compared with neutral priming condition) will lead to less negative emotional experiences, since priming inhibition word would induce effective AER to those negative pictures. Previous studies showed that automatic or implicit emotion regulation in the conscious level is associated with activity in OFC, amygdala, mPFC, and vlPFC (Hallam et al., 2015; Lieberman et al., 2007; Yoon et al., 2015). Meanwhile, the bilateral dorsolateral prefrontal cortex (dlPFC) is involved in effortful executive processing (Golkar et al., 2012; Vanderhasselt, Raedt, Baeken, Leyman, & D’haenen, 2006). Based on existing evidences and the neural model of AER proposed by Phillips et al. (2008), we hypothesized that the different neural correlates associated with the AER at the implicit and explicit level lie in the prefrontal cortex regions (e.g., dlPFC and vlPFC) and some limbic regions (e.g., amygdala). To this end, the exploratory analyses of regions of interest (ROIs) were conducted (e.g., explicit vs. implicit; neutral vs. inhibition).

## Method

### Participants

Thirty-eight college students participated in this experiment. They were all right handed with normal or corrected-to-normal vision. None had any history of psychiatric illness, brain disease, or head trauma. Data of four participants had to be excluded from further statistical analysis, due to their severe head motion (translation >3 mm or rotation >3°). The remaining 34 participants (18 females) aged from 18 to 25 years (mean ± *SD* = 20.9 ± 2.4 years).

This study was approved by the Ethics Committee of Center for Cognition and Brain Disorders in Hangzhou Normal University. All the participants gave written informed consent before the experiment.

### Materials

A total of 20 Chinese two-character verbs were used as priming stimuli. Half of these words were inhibition words (e.g., 克制 [*ke zhi*, meaning *restrain*]), and another half were neutral words (e.g., 询问 [*xun wen*, meaning *inquire*]). The control sense of these words were rated by three experts in the field of affective research on a 9-point Likert scale (1 representing *no control* and 9 representing *greatest control*). The control sense of the inhibition words (7.8 ± 0.6) was significantly greater than that of the neutral words (2.1 ± 0.4; *p* < .001). The emotional valence of these words were also rated by 20 college students who did not participate in the formal experiment on a 9-point Likert scale (1 representing *most negative* and 9 representing *most positive*). There was no significant difference in the emotional valence between the inhibition words (4.5 ± 1.0) and neutral words (5.1 ± 0.5; *p* = .114). Word frequency (mean_inhibition = 2743.8, range = 227–12472 per million; mean_neutral = 1058.2, range = 227–3023 per million; Modern Chinese Frequency Dictionary, 1986) and stroke numbers (mean_inhibition = 15.2 ± 2.3, mean_neutral = 17.9 ± 4.5) were also matched between inhibition words and neutral words (all *ps* > .050).

A total of 160 negative pictures from the Chinese Emotion Picture System (CAPS; Bai, Ma, Huang, & Luo, 2005) were selected as the experiment materials. The valence of all the pictures was either equal to or less than 3 according to the rating results of the CAPS system (1 representing the *most negative* and 9 representing the *most positive*). These pictures were randomly allocated to four groups, each containing 40 pictures. There was no significant difference in the emotional valence (2.4 ± 0.4, 2.4 ± 0.4, 2.4 ± 0.4, and 2.4 ± 0.4) and arousal (5.5 ± 0.8, 5.7 ± 0.9, 5.7 ± 0.9, and 5.6 ± 0.8; according to the CAPS rating results: 1 = *calm*, 9 = *excited*) across the four groups (all *p*s > .050).

In the present study, a priming word was presented simultaneously with a negative picture, and each word was presented eight times. Across the word types (inhibition vs. neutral) and perceptual levels (explicit vs. implicit), there were four conditions: implicit inhibition condition (II), implicit neutral condition (IN), explicit inhibition condition (EI), and explicit neutral condition (EN). The four groups of negative pictures were randomly allocated into the above four conditions.

### Procedure

Before the fMRI experiment, a perceptual level measurement was performed to obtain the thresholds of the implicit and explicit perceptual level for each participant. Through this way, the presentation time of the priming words in the fMRI experiment was determined.

#### Perceptual level measurement

The implicit and explicit perceptual level of each participant was measured by adopting the subjective and objective threshold paradigm developed by Marcel (1983). Participants were told that they would complete a forced task which contained 9 blocks with 10 trials included in each block. Each trial consisted of four components. First, a fixation was displayed in the center of the screen for 500 ms. Then a two-character Chinese word appeared in the center of the screen for a certain duration time, ranging from 150 ms to 16.7 ms downwards with an interval of 16.7 ms (i.e., the screen refresh time). Then a mask which is an image consisting of lines of meaningless sequence of letters in a dark gray background: “
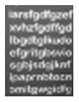
”) was displayed for 500 ms. Finally, a 5000-ms forced task was followed, in which the participants must select one out of two words that she or he thought to have seen it before the presence of mask. Then the correct rate (CR) of the task completion was calculated. In this study, the threshold of the implicit perceptual level was defined as the minimal duration of the stimulus when the CR of the forced-choice task was above 60% and stable (Marcel, 1983). The threshold of the explicit perceptual level was defined as the duration of the stimulus when the CR was equal to 100%. When the words were presented at 3000 ms, the CR for all the participants reached 100%, so 3000 ms was determined as the threshold of the explicit perceptual level. By this procedure, each participant’s threshold of the implicit perceptual level was finally obtained, which is either 33 ms or 50 ms. All the words adopted in this screening process were not used in the later fMRI experiment.

#### fMRI procedure

**Figure 1.**
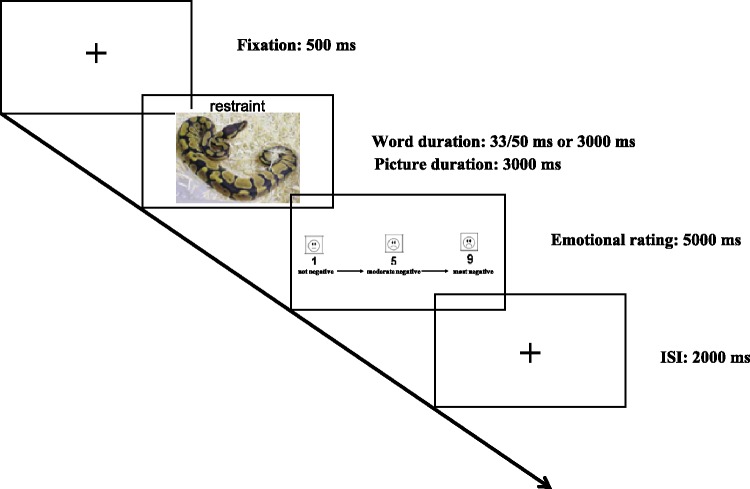
The experimental paradigm. ISI = interstimulus interval.

The experiment was an event-related design, containing five runs. Each run began with 5 *dummy* volumes (with a fixation in the screen) to allow for T1 equilibration. Besides the 10 s *dummy* images, each run was composed of four experimental conditions (II, IN, EI, and EN), each including eight trials. For each trial, a 500 ms “+” was presented first. The display of a 3000-ms negative picture combined with inhibition words or neutral words randomly then followed, while the words might disappear after 33 ms or 50 ms which was the threshold of the participant’s implicit perceptual level, or after 3000 ms at the explicit perceptual level. The participants were required to make emotional valence rating of negative pictures that they had just viewed on a 9-point Likert scale (1 = *not negative*, 5 = *moderate negative*, 9 = *most negative*), without mentioning the role of words above pictures. Responses were given via pressing a button 1 to 9 times within 5000 ms. Then a 2000-ms fixation was presented for a break (cf. Hallam et al., 2015; Ochsner et al., 2004). The whole process of a trial was presented in Figure 1. The sequences of runs and experimental conditions were counterbalanced across the participants.

Before the fMRI scanning, several practice trials were provided to the participants to familiarize themselves with the experiment. After the experiment, the participants were inquired about whether they saw the words above the pictures. All the participants reported “Not all of the pictures have a word at the top.”

### Data Acquisition

Image scanning was conducted on a GE 3.0 T scanner with a standard head coil. The participants were positioned supine inside the scanner, and all visual stimuli were projected onto the screen. Forty-three axial slices covering the whole brain were acquired using a T2*-weighted gradient echo-planar imaging pulse sequence (TR [time of repetition] = 2000 ms, TE [time of echo] = 30 ms, flip angle = 90°, acquisition matrix = 64 × 64, FOV [field of view] = 220 mm × 220 mm, slice thickness = 3.2 mm, no gap) for the functional scans. A structural three-dimensional image was acquired after the five functional runs.

### Data Analysis

#### Behavioral data analysis

The behavioral data were the participants’ subjective emotion ratings of negative pictures during the experiment. Behavioral data analysis was carried out using SPSS 17.0. The data were demonstrated to be normally distributed by Kolmogorov–Smirnov test and homogeneous in variance by Bartlett’s test. A 2 (Perceptual Level: implicit, explicit) ×2 (Priming Type: inhibition, neutral) repeated measures analysis of variance (ANOVA) on emotional rating scores were performed.

#### fMRI data analysis

SPM8 (Welcome Department of Cognitive Neurology, London, UK; http://www.fil.ion.ucl.ac.uk/spm/) based on the MATLAB 12 software package (The Math Works Inc., Natick, MA) was used for fMRI data preprocessing and statistical analyses. For each run, the first five dummy images were cast-off. The functional images were realigned to the first volume to correct for head motion, spatially normalized to the echo-planar imaging template with 2 × 2 × 2 mm^3^ spatial resolution and then spatially smoothed with an 8-mm FWHM Gaussian filter.

The general linear model was used to estimate the condition effect with canonical hemodynamic response function for each subject. The data were globally scaled and high-pass filtered at 128 s. Individual results were acquired by defining four effects of interest for each subject: (a) II: implicit perception level with inhibition priming word, (b) IN: implicit level with neutral word, (c) EI: explicit level with inhibition word, and (d) EN: explicit level with neutral word. Group-averaged effects were computed with a second-level random-effect model. Effects of interest were calculated for the direct contrast between implicit and explicit perception levels, inhibition priming and neutral priming conditions.

To investigate the interaction effect in the brain activity between the perception levels (implicit vs. explicit) and priming types (inhibition vs. neutral), we carried out exploratory analyses of the ROIs. According to the neural network of AER proposed by Phillips et al. (2008) and the crucial regions found to be associated with emotion regulation in the existing literature, the following areas were selected as ROIs: bilateral ACC (left: *x* = −4, *y* = 38, *z* = 28; right: *x* = 4, *y* = 38, *z* = 28), bilateral OFC (left: *x* = −26, *y* = 24, *z* = −22; right: *x* = 26, *y* = 24, *z* = −22), dorsomedial prefrontal cortex (*x* = −2, *y* = 52, *z* = 40), left dlPFC (*x* = −42, *y* = 10, *z* = 26), left vlPFC (*x* = −58, *y* = 24, *z* = 6), right parahippocampal gyrus or hippocampus (x = 29, y = −23, z = −11), and left amygdala (*x* = −18, *y* = 0, *z* = 18). The ROI masks were created as spheres with a 6 mm radius, centered on MNI coordinates identified in previous research (Banks, Eddy, Angstadt, Nathan, & Phan, 2007; Das et al., 2005; Grimm et al., 2009; Hallam et al., 2015; Williams et al., 2001; Yoon et al., 2015). With reference to the thresholds previously adopted (Koch et al., 2007), a voxel-wise intensity threshold (*p* < .001, uncorrected) and a spatial extent threshold of cluster size greater than 40 voxels were used for the brain regions selected a priori.

The percent signal changes of the aforementioned ROIs in different conditions were extracted, using the MarsBaR software (http://marsbar.sourceforge.net/). Extracted data were then subjected to ANOVA analysis using SPSS 17.0.

## Results

### Behavioral Results

**Figure 2.**
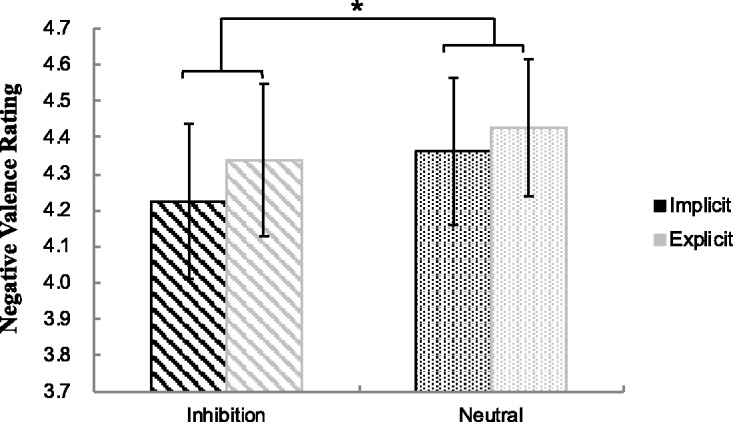
Mean emotional valence rating in each condition. The error bars show standard errors (*SE*). *p < .05.

Results of a 2 (Perceptual Level: implicit, explicit) × 2 (Priming Type: inhibition, neutral) repeated measures ANOVA on emotional rating scores revealed that there was a significant main effect of priming type, *F*(1, 33) = 5.41, *p* < .050, $\eta_{p}^{2}$= .141. The main effect of perceptual level, *F*(1, 33) = 2.70, *p* = .110, and interaction effect between priming type and perceptual level, *F*(1, 33) = 0.31, *p* = .584, were not significant (see Figure 2). Such results suggested that the participants experienced less negative emotion in inhibition priming condition than in neutral priming condition.

### fMRI Results

**Figure 3.**
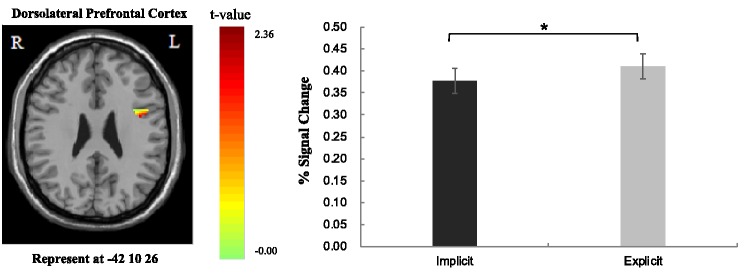
The main effect of perceptual level in the left dlPFC: explicit > implicit. The error bars show standard errors (*SE*). *p < .05.

**Figure 4.**
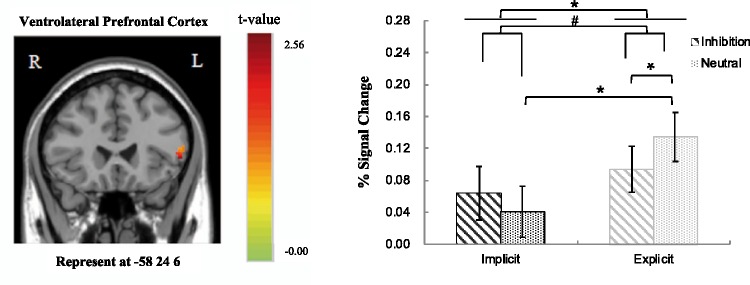
The main effect of perceptual level (explicit > implicit) and marginally significant interaction effect between perceptual level and priming type in the left vlPFC. The error bars show standard errors (*SE*). ^#^*p* < 0.1. *p < .05.

Group effect of the comparisons between implicit and explicit perception levels showed that only the left vlPFC and dlPFC among the selected ROIs were found to be activated in the contrast. The 2 (Perceptual Level: implicit, explicit) × 2 (Priming Type: inhibition, neutral) repeated ANOVA analysis based on the fMRI percent signal changes also showed a significant main effect of perception level in the left vlPFC, *F*(1, 33) = 8.15, *p* < .050, $\eta_{p}^{2}$ = .198, and left dlPFC, *F*(1, 33) = 4.14, *p* < .050, $\eta_{p}^{2}$ = .111. Greater activations in both regions were elicited in the explicit perceptual level than in the implicit perceptual level (see Figures 3 and 4). For the comparisons between neutral and inhibition conditions, only the left dlPFC among the selected ROIs was found to be activated in the contrast. The ANOVA analysis of the percent signal changes also showed a significant main effect of priming type in the left dorsolateral PFC, *F*(1, 33) = 17.17, *p* < .001, $\eta_{p}^{2}$ = .342. Greater activation in this region was found in response to neutral priming than to inhibition priming condition. There was a marginally significant interaction effect in the left vlPFC, *F*(1, 33) = 2.88, *p* = .099, $\eta_{p}^{2}$ = .080. Results of the multiple comparisons showed that there was greater activation in this area under the neutral priming condition as compared with inhibition priming condition at explicit perceptual level (*p* < .050) and a significant activation difference between the EN and IN trials (*p* < .050). No other selected ROIs were found to be associated with the main and interaction effects of the ANOVA analysis.

## Discussion

This study aimed at two specific research questions: Can inhibition word effectively prime AER, and what differences may there be in the activation of certain brain regions between the implicit and explicit perceptual levels during AER. The findings of this study indicate that the participants experienced less negative emotion in the inhibition words priming condition in comparison with the neutral words priming condition. Significant differences were also found in the left vlPFC and left dlPFC at different perception levels in the AER process.

It is important to note in this study that inhibition words are effective in initiating AER to help participants regulate their negative emotions since they gave lower negative emotion ratings to the negative pictures associated with the inhibition words than those with neutral words. As far as we know, previous research has shown that different priming techniques can serve as a useful strategy to manipulate implicit regulatory processes (Yang et al., 2015). For example, Mauss et al. (2007) used sentence-construction task to prime AER. When the participants were asked to sequence four words containing emotional control terms into one sentence, they successfully activated their AER process. Likewise, sentence unscrambling task containing inhibition words has also been found to be effective to prime individual’s unconscious emotion regulation (Raes, Dewulf, Heeringen, & Williams, 2009). Different from the priming techniques of those previous studies, the present study only adapted the priming strategy by presenting inhibition words in order to prime the participants’ AER. The fact that inhibition words priming can effectively manipulate automatic regulatory process offers supporting evidence to the findings of previous studies that AER can be independent of cognitive resource constraints (Carstensen & Mikels, 2005).

More importantly, we also found that there is a difference of neural underpinnings of AER between explicit and implicit perceptual levels in the present study. The left vlPFC and left dlPFC regions reveal greater activation at the explicit perceptual level relative to implicit perceptual level. It is evident in the present study that the participants have had enough time to process the priming words at the explicit level (being presented for 3000 ms) automatically as compared with the implicit priming condition (being presented only for 33 or 50 ms). Despite the assumption that the right vlPFC is a strong candidate of brain region being central to exert self-control (Cohen et al., 2013), a few scholars contend that activity of the vlPFC in healthy participants is sometimes bilateral (Aron et al., 2003; Chambers et al., 2007). Thus, not to our surprise, the findings of this study have confirmed to some extent the role of the left vlPFC in self-control at an explicit perceptual level.

Moreover, prior research has identified the association between the activation in dlPFC and effortful emotion regulation strategies (Banks et al., 2007). Recently, Braunstein, Gross, and Ochsner (2017) proposed an emotion regulation framework based on two orthogonal dimensions (i.e., the nature of the emotion regulation goal and the nature of the emotion change process), which posits the activation in dlPFC especially in association with explicit automatic regulation. The fact that we found greater activation of dlPFC at the explicit level than at implicit level of perception, regardless of the priming word type (i.e., neutral vs. inhibition words) seems to have provided some empirical support to Braunstein et al.’s (2017) theoretical framework which suggests the role of the left dlPFC involved in the explicit AER process.

Furthermore, we found a decreased activation in the left dlPFC under the inhibition word priming condition (contrary to the neutral word priming condition). The dlPFC was another prefrontal brain region often activated in the tasks requiring self-control (Cohen et al., 2013). In addition, the left dlPFC was a region crucially involved in cognitive labeling (e.g., appraisal) of negative emotional stimuli (Hariri, Bookheimer, & Mazziotta, 2000; Hariri, Mattay, Tessitore, Fera, & Weinberger, 2003; Taylor, Phan, Decker, & Liberzon, 2003) and played the role of inhibition control. In the present study, the participants were not explicitly required to view and process the priming word, thus might result in the decreased activity in the left dlPFC in the inhibition condition compared with neutral condition. However, future research should be done to further explore the role of dlPFC in the AER process.

Apart from the earlier significant findings, we also found a marginally significant interaction effect in the left vlPFC. In other words, the greater activation of left vlPFC was observed when compared neutral words with inhibition words at the explicit perceptual level, and greater activity of left vlPFC was associated with neutral priming condition at explicit level when compared with implicit level. In the present study, the emotional valence of word materials was matched between inhibition words and neutral words. If the specificity of vlPFC was associated with the affective valence of stimuli, we should not find any activation differences in the left vlPFC. The activity of left vlPFC was weaker in the EI condition (contrary to EN condition) during the AER process, suggesting that people can fulfill inhibition control effectively in the inhibition priming condition at the explicit level. The right vlPFC was found to be implicated in inhibitory control (Aron et al., 2014) and participated in the implicit form of emotion control (Cohen et al., 2013), and the left vlPFC can also play an independent role in inhibitory control during AER. Yoon et al. (2015) revealed that a nonconscious form of emotion regulation was mediated by the right vlPFC by using facial expression to prime the implicit regulation of negative photos. We manipulated the automatic regulation of negative pictures by adopting inhibition words as priming. These differences in tasks and stimuli might contribute to the different results in our study and the Yoon et al.’s study.

In addition, we did not find significant differences between implicit inhibition and explicit inhibition conditions, and between implicit inhibition and implicit neutral conditions. The dlPFC was associated with self-control (Cohen et al., 2013) and explicit AER (Banks et al., 2007; Braunstein et al., 2017). Thus, it is reasonable to expect that the dlPFC was involved in the comparisons between the implicit and explicit inhibition conditiions. However, we did not find significant different activity of the dlPFC for the comparison. Such nonsignificance results may be due to the methodological difference between the present study and those previous studies. In our study, the inhibition words were only presented above the negative pictures, and the participants were not required to explicitly process the words, thus no brain regions responded differently in the implicit and explicit level. Similarly, no significant brain regions was found in the comparisons of inhibition and neutral conditions at the implicit level, as the brief and passive processing of the words may lead to weak brain response in both conditions. It is also worth noting that although the use of 10.5 s trial length and 2 s TR constituted an *offset TR* method of jittering, additional jittering of the length of the interstimulus interval may lead to better statistical efficiency or power (Dale, 1999). Thus, future study should be deliberately designed to have jittered or randomized interstimulus interval from trial to trial.

In conclusion, the present study reveals that inhibition words can effectively prime AER, leading to less negative emotions experienced by individuals. The neural differences underlying the AER process at the explicit and implicit perceptual levels were found in left vlPFC and left dlPFC regions, suggesting that these two regions mainly participate in self-control. The findings of our study could have important implications for practice. The fact that inhibition words prime AER could be adopted to improve an individual’s adaptive ability and reduce the risk of emotional disorders.
